# Laparoscopic decortication with harmonic ultrasonic energy for large recurrent renal cyst: a case report and literature review

**DOI:** 10.1093/jscr/rjag657

**Published:** 2026-07-31

**Authors:** Dharmendra Kumar Pipal, Gaurav Gupta, Swati Prasad, L Rajesh Kannan, Alan Philips, Nikhil B, Shashikant Sai, Raja Murtaza Aaqib, Vikas Prasad, Pearl Yadav, Divya Mishra, Twvisham Srivastav, Nischit B Murgod

**Affiliations:** Department of General Surgery, All India Institute of Medical Sciences, Gorakhpur, Uttar Pradesh, India; Department of General Surgery, All India Institute of Medical Sciences, Gorakhpur, Uttar Pradesh, India; Department of General Surgery, All India Institute of Medical Sciences, Gorakhpur, Uttar Pradesh, India; Department of General Surgery, All India Institute of Medical Sciences, Gorakhpur, Uttar Pradesh, India; Department of General Surgery, All India Institute of Medical Sciences, Gorakhpur, Uttar Pradesh, India; Department of General Surgery, All India Institute of Medical Sciences, Gorakhpur, Uttar Pradesh, India; Department of General Surgery, All India Institute of Medical Sciences, Gorakhpur, Uttar Pradesh, India; Department of General Surgery, All India Institute of Medical Sciences, Gorakhpur, Uttar Pradesh, India; Department of General Surgery, All India Institute of Medical Sciences, Gorakhpur, Uttar Pradesh, India; Department of General Surgery, All India Institute of Medical Sciences, Gorakhpur, Uttar Pradesh, India; Department of General Surgery, All India Institute of Medical Sciences, Gorakhpur, Uttar Pradesh, India; Department of General Surgery, All India Institute of Medical Sciences, Gorakhpur, Uttar Pradesh, India; Department of General Surgery, All India Institute of Medical Sciences, Gorakhpur, Uttar Pradesh, India

**Keywords:** simple recurrent renal cyst, laparoscopic decortication, harmonic device, sclerotherapy

## Abstract

Simple renal cysts are typically benign; however, large or recurrent cysts can cause chronic pain, abdominal distension, and renal compression, requiring intervention. We present a case of a 46-year-old female patient with persistent back pain and abdominal distension found on computed tomography to have a large left upper pole renal cyst measuring $8\times 10$ cm with significant parenchymal compression and impaired renal function. Percutaneous aspiration with sclerotherapy was initially attempted but the cyst recurred within one month. Transperitoneal laparoscopic decortication was subsequently performed using Harmonic scissors to excise the cyst wall. The procedure was completed in 60 minutes without intraoperative or postoperative complications, with uneventful patient recovery. This case demonstrates that laparoscopic decortication with harmonic energy is a safe, effective, and minimally invasive approach for managing large, symptomatic, and recurrent simple renal cysts.

## Introduction

Simple renal cysts are common, affecting ~12% of the general population, and tend to increase in both number and size with advancing age [1]. Most cases remain asymptomatic and require no intervention. However, symptomatic cysts may present with chronic flank pain, haematuria, infection, or, rarely, malignant transformation. Percutaneous aspiration, with or without sclerotherapy, is frequently employed as first-line treatment; however, recurrence rates are notably high following these approaches [2]. Although less commonly utilized, laparoscopic decortication offers superior efficacy, with higher success rates, reduced intraoperative blood loss, and shorter operative time compared to percutaneous techniques.

## Case report

**Figure 1:**
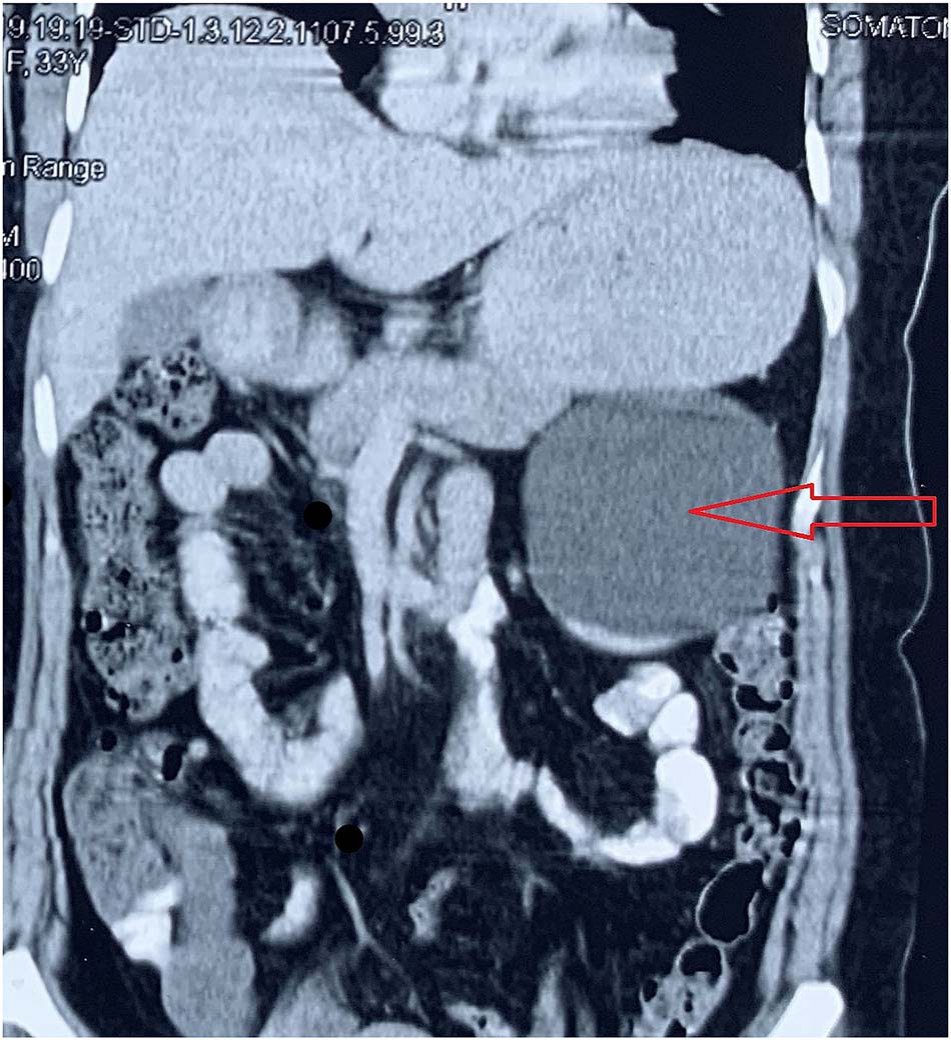
Renal cyst and compromised renal cortex depicted by arrow.

**Figure 2:**
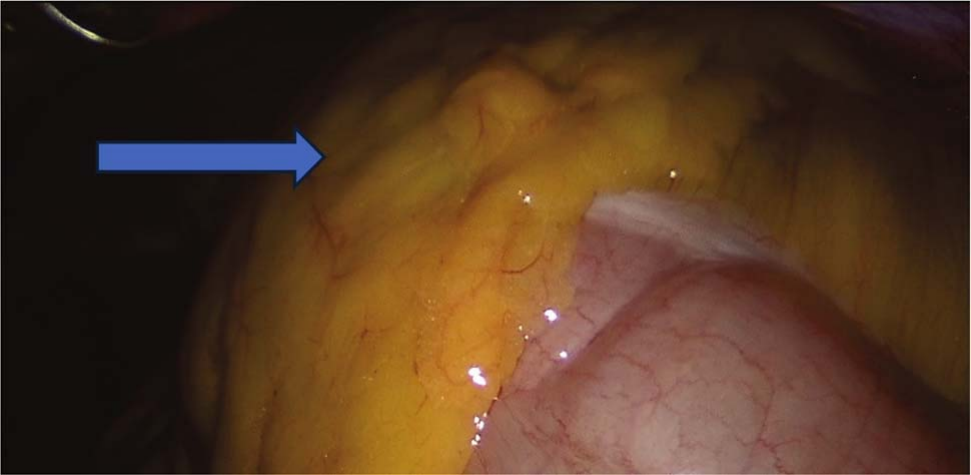
Intraoperative image showing the covered renal cyst by gut.

**Figure 3:**
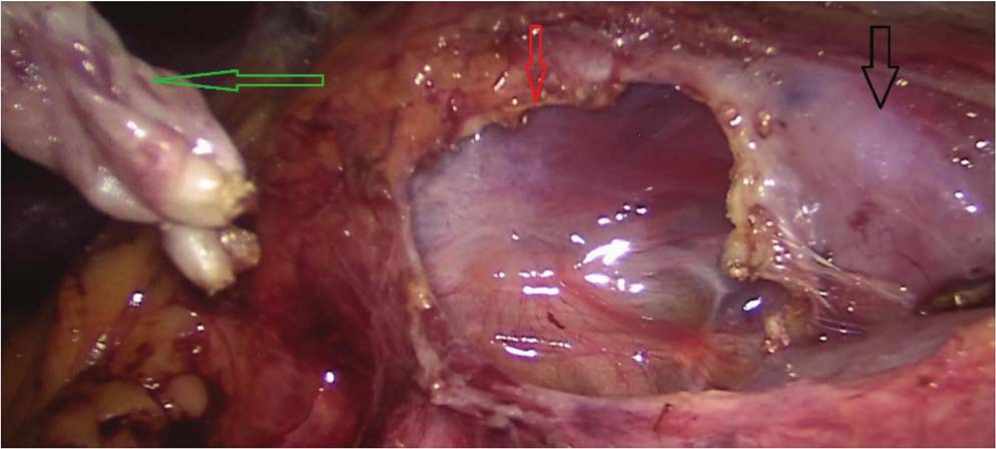
Deroofed cystic cavity after excising the cyst wall with the help of an ultrasonic energy device (red arrow depicting cyst at the upper pole of the left kidney, green arrow depicting the excised cyst wall and black arrow depicting the compromised renal tissue).

A 46-year-old female with no significant medical history presented to the surgical clinic at All-India Institute of Medical Sciences, Gorakhpur, India, with a six-month history of progressive dull back pain (VAS 40 mm) and abdominal fullness. Computed tomography (CT) identified a 8 × 10 cm left simple renal cyst (Fig. 1), and a Tc-99 m radionuclide scan confirmed impaired relative renal function at 30%. Initial management via ultrasound-guided aspiration of 1000 ml of clear fluid and 20% hypertonic saline sclerotherapy failed, with follow-up imaging at 2 months revealing an 8 × 10 cm recurrence alongside symptomatic relapse. Consequently, the patient underwent transperitoneal laparoscopic decortication in the lateral decubitus position. Intraoperatively, the massive upper-pole cyst was found significantly compressing the renal parenchyma and visibly displacing the splenic flexure (Fig. 2). After aspirating 1500 ml of cyst fluid, the cyst wall was circumferentially excised using a Harmonic ultrasonic device (Fig. 3). The procedure was completed in 60 minutes with minimal blood loss, and the patient was discharged on postoperative Day 5 following an uneventful recovery. Histopathology confirmed a benign cyst wall, and a 6-month follow-up CT demonstrated complete symptomatic resolution with no evidence of recurrence.

## Discussion

While simple renal cysts occur across all age groups, their prevalence and dimensions increase with advancing age [3, 4]. Although the majority remain asymptomatic and require no intervention, surgical management is indicated for complications such as chronic pain, infection, haematuria, renal function impairment, or cyst rupture. Percutaneous aspiration, with or without sclerotherapy, is often utilized as a first line treatment [5, 6]; however, its efficacy is frequently limited by high recurrence rates, often necessitating repeated procedures [7]. In the present case, initial percutaneous drainage and sclerotherapy were performed but resulted in a rapid symptomatic and radiological recurrence. Numerous studies have highlighted the superior outcomes of laparoscopic decortication for the management of renal cysts, citing high success rates and significantly lower recurrence compared to other modalities [8–10]. Specifically, while the success rate for percutaneous sclerotherapy is ~55%, laparoscopic decortication consistently achieves success rates exceeding 95% [11]. Within the framework of the Bosniak classification system, laparoscopic decortication is considered a preferred therapeutic approach for symptomatic Grade I simple renal cysts. Suspicious cystic renal lesions require either total or partial nephrectomy; renal cell carcinoma has been incidentally discovered during laparoscopic decortication, necessitating subsequent nephrectomy [12]. Comprehensive preoperative evaluation is essential for Bosniak Grade II cysts and higher. A documented case revealed an intraoperatively discovered urinary tract connection, contradicting the diagnostic criterion of uncomplicated cysts; suture closure of the fistula was performed. Preoperative retrograde urethrography or percutaneous puncture may be necessary to exclude urinary tract communication and ensure surgical safety.

The transperitoneal laparoscopic approach was selected for this case due to the cyst’s large size, necessitating an additional port for optimal visualization and manipulation. Although retroperitoneal laparoscopy is associated with reduced postoperative discomfort compared to the transperitoneal technique [13, 14], the transperitoneal approach offers superior visualization and is preferred for ventral renal cysts requiring extensive mobilization, despite the increased risk of bowel injury and postoperative ileus. An ultrasonic harmonic device was employed for cyst decortication in this case. Various energy sources are utilized in laparoscopic surgery, including monopolar devices, argon beam coagulators [7], and sealing devices. Sealing devices have been reported to improve safety and reduce operative time [4, 8]; however, Tuncel *et al*. [15] demonstrated that conventional monopolar devices remain a safe and cost-effective alternative for symptomatic simple renal cysts. While cost considerations are important, the harmonic sealing device offers enhanced safety and shorter operative duration, making it a viable option when balancing efficacy, patient safety, and procedural efficiency.

In conclusion, most simple renal cysts remain asymptomatic and require surveillance; however, symptomatic cysts impairing renal function necessitate intervention to prevent progressive parenchymal damage. Laparoscopic decortication offers a definitive, minimally invasive approach superior to image-guided aspiration, which has high recurrence rates requiring repeated procedures. The harmonic ultrasonic device is an ideal energy source for decortication, ensuring complete cyst resolution, preserving renal function, minimizing tissue trauma, and reducing operative time and postoperative morbidity.

## Data Availability

Will be given when asked.

## Declaration

AI help: Used QuilBot, AI Fiesta for writing and correcting grammar of the report.

## References

[1] Terada N, Ichioka K, Matsuta Y et al. The natural history of simple renal cysts. J Urol 2002;167:21–3. 11743266

[2] Wolf JS Jr . Evaluation and management of solid and cystic renal masses. J Urol 1998;159:1120–33. 9507815

[3] Dunn MD, Clayman RV. Laparoscopic management of renal cystic disease. World J Urol 2000;18:272–7. 10.1007/pl00007076 11000310

[4] Topaktas R, Akkoc A, Altin S et al. Effectiveness of harmonic scalpel in laparoscopic treatment of simple renal cyst. J Pak Med Assoc 2018;68:1124–8. 30317318

[5] Eissa A, El Sherbiny A, Martorana E et al. Non-conservative management of simple renal cysts in adults: a comprehensive review of literature. Minerva Urol Nefrol 2018;70:179–92. 10.23736/S0393-2249.17.02985-X 29611673

[6] Cheng D, Amin P, Ha TV. Percutaneous sclerotherapy of cystic lesions. Semin Intervent Radiol 2012;29:295–300. 10.1055/s-0032-1330063 24293802 PMC3577633

[7] Atug F, Burgess SV, Ruiz-Deya G et al. Long-term durability of laparoscopic decortication of symptomatic renal cysts. Urology 2006;68:272–5. 10.1016/j.urology.2006.03.009 16904433

[8] Erdem MR, Tepeler A, Gunes M et al. Laparoscopic decortication of hilar renal cysts using LigaSure. JSLS 2014;18:301–7. 10.4293/108680813X13753907291558 24960497 PMC4035644

[9] Efesoy O, Tek M, Bozlu M et al. Comparison of single-session aspiration and ethanol sclerotherapy with laparoscopic de-roofing in the management of symptomatic simple renal cysts. Turk J Urol 2015;41:14–9. 10.5152/tud.2015.77675 26328192 PMC4548655

[10] Nalagatla S, Manson R, McLennan R et al. Laparoscopic decortication of simple renal cysts: a systematic review and meta-analysis to determine efficacy and safety of this procedure. Urol Int 2019;103:235–41. 10.1159/000497313 30889610

[11] Bas O, Nalbant I, Can Sener N et al. Management of renal cysts. JSLS 2015;19:e2014.00097. 10.4293/JSLS.2014.00097 PMC4376217 25848184

[12] Santiago L, Yamaguchi R, Kaswick J et al. Laparoscopic management of indeterminate renal cysts. Urology 1998;52:379–83. 10.1016/s0090-4295(98)00213-1 9730447

[13] Ozcan L, Polat EC, Onen E et al. Comparison between retroperitoneal and transperitoneal approaches in the laparoscopic treatment of Bosniak type I renal cysts: a retrospective study. Urol J 2015;12:2218–22. 26341761

[14] Wright JL, Porter JR. Laparoscopic partial nephrectomy: comparison of transperitoneal and retroperitoneal approaches. J Urol 2005;174:841–5. 10.1097/01.ju.0000169423.94253.46 16093966

[15] Tuncel A, Aydin O, Balci M et al. Laparoscopic decortication of symptomatic simple renal cyst using conventional monopolar device. Kaohsiung J Med Sci 2011;27:64–7. 10.1016/j.kjms.2010.09.002 21354520 PMC11916431

